# Synergistic antitumor effects of combined pembrolizumab and anlotinib via EGFR inhibition and tumor microenvironment remodeling in high-grade serous ovarian cancer

**DOI:** 10.3389/fonc.2026.1880902

**Published:** 2026-10-19

**Authors:** Xiaoyang Yin, Longxiao Zhang, Yidan Mu, Guangping Li, Xiaoyu Li, Desheng Zheng, Xuwei Tian, Reziya Emir, Yu Liang, Peng Zhao, Man Jiang

**Affiliations:** 1 Department of Pharmacy, The Affiliated Hospital of Qingdao University, Qingdao, China; 2 Department of Radiation Oncology, The Affiliated Hospital of Qingdao University, Qingdao, China; 3 Department of Pathology, The Affiliated Hospital of Qingdao University, Qingdao, China; 4 School of Pharmacy, Qingdao University, Qingdao, China; 5 School of Information and Software Engineering, University of Electronic Science and Technology of China, Chengdu, China; 6 School of Computer Science and Software Engineering, Southwest Petroleum University, Chengdu, China; 7 The First People’s Hospital of Kashi, Kashgar, China; 8 Department of Oncology, the First People’s Hospital of Kashgar Prefecture, Kashgar, China

**Keywords:** anlotinib, drug synergy, HGSOC, PDX, pembrolizumab

## Abstract

**Background:**

High-grade serous ovarian cancer (HGSOC) remains difficult to treat, particularly in platinum-resistant or recurrent cases. Despite ongoing clinical trials investigating pembrolizumab combined with anlotinib, the synergistic mechanisms of this regimen are poorly understood.

**Methods:**

This study constructed humanized patient-derived xenograft models via implanting HGSOC tumor cells into NSG mice and subsequently infusing human peripheral blood mononuclear cells to dissect synergistic mechanisms.

**Results:**

Relative to controls and pembrolizumab monotherapy, combination therapy raised the fractions of CD4^+^ T cells and CD8^+^/NK cells while decreasing myeloid-derived suppressor cell abundance. Enhanced cell-cell interactions from monocyte and CD8^+^/NK-cell senders to epithelial targets were observed in the combination group. Upregulation of GRN-SORT1 signaling mediated communication between myeloid subclusters 1, 7 and epithelial subclusters 0, 2, 3, whereas CCL7-CCR1 signaling was activated between myeloid subcluster 7 and lymphocyte subcluster 0. A decreased Treg proportion was also achieved under combined treatment. Multicolor immunofluorescence of patient HGSOC tissues showed close spatial contacts between CD14^+^CCL7^+^ monocytes and CD8^+^CCR1^+^ T cells, and CCL7 as well as CCR1 expression positively correlated with intratumoral CD8^+^ T-cell infiltration.

**Conclusion:**

These findings suggest that pembrolizumab and anlotinib may synergize through both limiting tumor-promoting pathways and enhancing anti-tumor immunity. This combinatorial regimen may represent a promising therapeutic strategy for improving clinical outcomes in HGSOC patients.

## Introduction

High-grade serous ovarian cancer (HGSOC) is the most aggressive and prevalent subtype of epithelial ovarian cancer, characterized by high recurrence rates and poor prognosis (1). Despite advancements in treatment, especially targeted therapies and immune therapies, resistance to treatment and immune evasion remain major obstacles to improving patient outcomes (2). Numerous clinical trials have investigated the use of immunotherapies, specifically immune checkpoint inhibitors (ICIs) in ovarian cancer (OC), however, clinical trials evaluating ICIs as a monotherapy for OC have yielded disappointing outcomes (3). For this reason, studies have progressed to combining ICIs with other treatment options such as tyrosine kinase inhibitors (TKIs). Anlotinib is a multi-targeted TKI that inhibits VEGFR1/2/3, FGFR1/2/3, PDGFR-α, c-Kit, and Ret, and has demonstrated efficacy across various cancer types (4). Emerging studies have reported that anlotinib exhibits favorable antitumor activity and acceptable safety profiles in ovarian cancer (5, 6). Notably, combination of anlotinib with immunotherapy has shown enhanced anticancer effects compared to monotherapy (7). The underlying mechanism might be that VEGF has direct and indirect immune suppressive effects by hindering the tumor infiltration of immune effector cells (8, 9), and increasing the infiltration of regulatory T cells (Tregs), myeloid derived suppressor cells (MDSCs), and M2 macrophages into the TME (10–13). Combining anlotinib with immunotherapy overcomes these mechanisms, helping to overcome the limitation of the immunosuppressive TME in HGSOC and thus enhancing the therapeutic effect. The combination of anlotinib with ICIs showed promising treatment effect in HGSOC as well as other cancer types. FDA has approved combinations of anti-VEGF/VEGFR with anti-PD-1/PD-L1 agents in various cancer types including hepatocellular carcinoma and renal cell carcinoma. Promising clinical activity has been demonstrated in patients with gastric cancer and gastroesophageal junction adenocarcinoma (14). Two clinical trials have shown that combining PD-1/PD-L1 inhibitors with bevacizumab holds promise for patients with recurrent ovarian cancer (15, 16). And there is a phase Ib study of anlotinib combined with PD-L1, which shows promising antitumor activity and manageable toxicities in patients with platinum-resistant or refractory ovarian cancer (17). Yang et al. reported that PD-L1 directly interacts with VEGFR2, promoting angiogenesis and metastasis in ovarian cancer through the c-JUN/VEGFR2 signaling axis (18). However, despite these findings, the detailed multicellular interactions and candidate signaling axes that sustain this synergy have not yet been comprehensively characterized at the single-cell level.

Epidermal growth factor receptor (EGFR) is another classical example of tyrosine kinase receptors (TKRs) that transmits extracellular signals into the cell regulating key process such as proliferation, migration and survival. And studies have found that VEGF and EGFR pathways share common downstream signaling including PI3K/AKT and RAS/RAF/ERK pathways. What’s more, up-regulated EGFR signaling increased VEGF through hypoxia-independent mechanisms and elevated VEGF in turn contributes to the emergence of resistance to EGFR TKIs (19). Crucially, the activation of the EGFR pathway induces PD-L1 expression and other immunosuppressive factors, thereby reshaping the tumor microenvironment to accomplish evasion of the host antitumor immune response (20). Combining a VEGF inhibitor with pembrolizumab serves as a promising approach bridging anti-angiogenesis and immunotherapy to synergistically attenuate this oncogenic crosstalk. High expression of EGFR signaling is a common feature of multiple cancers and EGFR signaling is tightly controlled by a negative feedback mechanism involving ligand-induced internalization and lysosomal degradation, which ensures proper signal termination. This negative feedback mechanism, governed by ligand-induced lysosomal degradation of EGFR, ensures signal termination and counteracts the oncogenic and transforming role of EGFR (21–23).

In this study, we established a humanized patient-derived xenograft (PDX) model of HGSOC by incorporating a humanized immune system through the introduction of peripheral blood mononuclear cells (PBMCs) in to immunodeficient mice to assess the impact of pembrolizumab monotherapy and pembrolizumab combined with anlotinib on the TME. And we employed single-cell RNA sequencing (scRNA-seq) to characterize cell populations in different treatment groups, allowing for an in-depth understanding of how these therapies influence cellular composition and interactions within the tumor. Given the emerging role of tumor-associated myeloid cells in shaping the TME, we further explored their subclusters and interactions with epithelial cells as well as immune cells across treatment groups.

By single cell sequencing of PDX model, this study provides novel insights into the synergy effects of pembrolizumab and anlotinib combination therapy in HGSOC.

## Materials and methods

### Multicolor immunofluorescence

A total of 30 patient samples were analyzed by multicolor immunofluorescence. Representative images were selected and presented. All immunofluorescence experiments were performed with three independent technical replicates per sample to ensure reproducibility. Paraffin sections were deparaffinized, rehydrated, and subjected to antigen retrieval in pH 6.0 citrate buffer using a microwave oven. The slides were heated at medium power for 8 minutes, allowed to stand for 8 minutes, and then heated at medium-low power for 7 minutes. After retrieval, the slides were cooled naturally to room temperature and washed three times with PBS on a rocking platform for 5 minutes each. Tissue areas are outlined with a hydrophobic pen, and endogenous peroxidase is blocked using 3% hydrogen peroxide for 25 min. After washing, serum blocking is applied (10% rabbit serum for goat antibodies, 3% BSA for others, blocking at room temperature for 25 minutes), followed by incubation with the first primary antibody overnight at 4 °C. HRP-conjugated secondary antibody is added, then TSA fluorophore, each with PBS/TBST washes. Antibody stripping is performed between rounds with elution buffer (room temperature + 37 °C). The process is repeated for up to four antibodies. After final staining, DAPI is used for nuclear counterstaining, followed by autofluorescence quenching and rinsing. Slides are mounted with antifade medium. Fluorescent imaging is performed using appropriate filters or spectral scanning. Full-slide scanning is recommended for best results. Primary antibody used in the experiment include CCL7 (1:100, Cat. No. Anp13476, IPODIX), CD14 (1:400, Cat. No. 60253-1-ig, Proteintech), CCR1 (1:100, Cat. No. Anp14198, IPODIX), CD8 (1:400, Cat. No. 66868-1-ig, Proteintech). Tyramide signal amplification (TSA) kit used in the experiment include DAPI (Cat. No. G1012), CCL7 (iF555-Tyramide, Cat. No. G1233), CD14 (iF440-Tyramide, Cat. No. G1250), CCR1 (iF488-Tyramide, Cat. No. G1231), CD8 (iF647-Tyramide, Cat. No. G1232).

### PDX model

Tumor cells were obtained from the peritoneal effusion of a 52-year-old female HGSOC patient, who had experienced disease progression during platinum-paclitaxel therapy and exhibited intolerance to Olaparib and bevacizumab. The detailed clinicopathological and genomic characteristics of the tumor donor are summarized in **Supplementary Table 1**, and the histopathological confirmation of HGSOC is shown in **Supplementary Figure 1**. The tumor was passaged to next generation (P1) after reaching a volume of 500–800 mm^3^. The following experiments were carried on P1 generation mice cohort.

PBMCs (MileCell Biotechnology) were obtained from a healthy donor and injected intraperitoneally into the PDX mice at a concentration of 5×10^6^ cells per mouse. After injection, flow cytometry was conducted to analyze CD45+ cells to confirm whether the humanized immune environment was successfully established (**Supplementary Figure 2**).

### Single-cell transcriptome analysis

Tumor samples were harvested and dissociated into single cell solution using the SoloTM Tumor Dissociation Kit (JZ-SC-58201) and filtered through a 40 μm strainer. Cells were stained with Calcein AM and Draq7 dyes to assess the viability before being loaded into a BD Rhapsody cartridge for transcriptome capture. Libraries were sequenced in paired-end 150 bp mode on the NovaSeq platform (Illumina). The sequencing data were processed using the BD Rhapsody Whole Transcriptome Assay Analysis Pipeline (v1.8) to generate a single-cell expression matrix, with GRCh38 as the reference genome.

The filtered matrix data from each sample was imported into R and analyzed using the Seurat package (Version 5.1.0). For quality control, cells with fewer than 200 genes, more than 3,000 genes, or mitochondrial gene content exceeding 30% of the total gene count were excluded. The data was then normalized, and the top 2,000 variable genes were selected. After scaling, principal component analysis (PCA) and Uniform Manifold Approximation and Projection (UMAP) were performed. Cell clustering was conducted, and marker genes for each cluster were identified using the FindAllMarkers function. Finally, cell annotation was carried out based on the marker genes specific to each cluster. For differential analysis across cell clusters and treatment groups, the Wilcoxon rank-sum test was employed using the FindAllMarkers and FindMarkers functions in the Seurat package.

### Cell interaction analysis

Cell interaction among different cell types was done by CellChat package (Version 2.1.2) for scRNA-seq. The statistical significance of ligand-receptor pairs and signaling communication pathways across groups was evaluated using the permutation test embedded in the CellChat package. *P* < 0.05 was considered statistically significant.

### Spatial transcriptome analysis

Spatial transcriptome data was downloaded from GSE203612. The matrix data was imported into R using Load10X_Spatial function and then processed for normalization, quality control, dimensionally reduction and clustering. Marker genes of each cluster were calculated and used for cell type annotation.

### Data and code availability

Data and code used in the manuscript will be made available on request.

## Results

### scRNA-seq of HGSOC patients’ tumor cells derived PDX model with humanized immune environment

HGSOC patients’ tumor cells were subcutaneously implanted into NSG mice followed by intraperitoneal administration of PBMCs to create a humanized immune environment. Then the mice were randomly divided into 3 groups (n = 5): the pembrolizumab monotherapy group, the pembrolizumab and anlotinib combination group and control group. PDX mice in the control group were administered oral vehicle solution twice a day for 4 weeks, with treatment initiated 14 days after tumor implantation. And in the pembrolizumab monotherapy group, mice were given pembrolizumab intravenously at a dose of 10 mg/kg twice a week (24, 25). All treatments were initiated 14 days after tumor implantation and sustained for 4 weeks. In the pembrolizumab and anlotinib combination group, mice received pembrolizumab at the identical dose, administration frequency and schedule as the monotherapy group, alongside oral anlotinib at 0.5 mg/kg once daily. All interventions in the combination group also commenced 14 days post-tumor implantation and lasted for 4 weeks. The anti-tumor efficacy of each regimen is shown in **Supplementary Figure 3**.

We then applied scRNA-seq to tumor samples from the humanized PDX models. After quality control and filtering, a total of 28,422 cells were retained for downstream analysis, including 9,942 cells from the control group, 8,823 cells from the pembrolizumab group, and 9,657 cells from the pembrolizumab plus anlotinib group. By clustering and marker genes (**Figure 1C**) identification, cells were classified into epithelial cells, lymphocytes (CD4+ T cells, CD8+/NK cells), myeloid cells (monocytes, myeloid-derived suppressor cells), stromal cells (fibroblasts, macrophages, m2_macrophages) and endothelial cells (**Figure 1A**). Marker gene were calculated using FindAllMarkers function with a fold change larger than 0.5 and min.pct equals 0.25 (**Figure 1C**). The proportion of those cell types were varied among different treatment, indicating the impact of different treatment. For example, the pembrolizumab and anlotinib combination group showed numerically the highest proportions of CD4+ T cells and CD8+/NK cells and the numerically lowest proportion of myeloid-derived suppressor cells (**Figures 1B, D**). The top features identifying different cell types showed the characterization of each cell types (**Figure 1E**).

**Figure 1:**
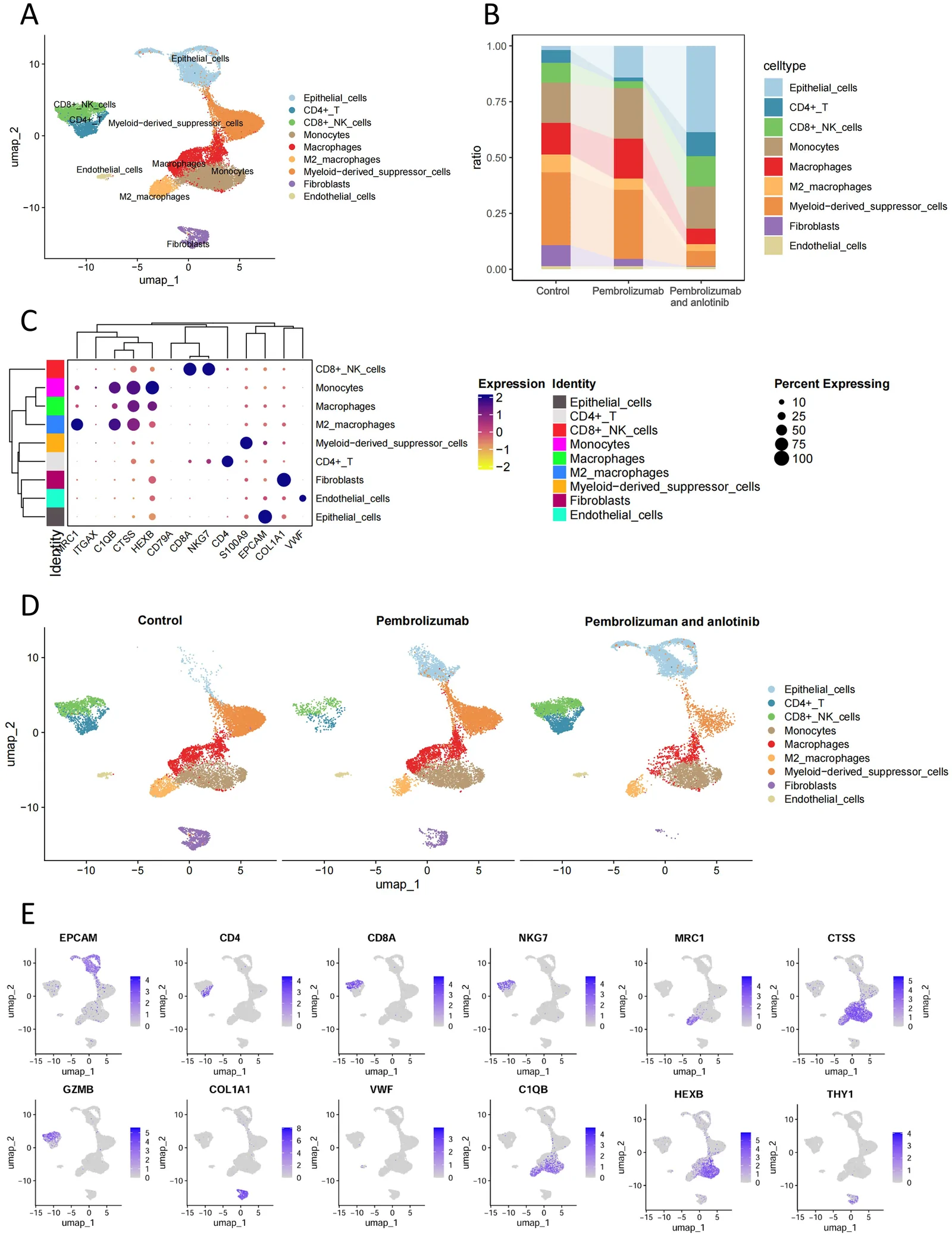
Overview of single-cell transcriptomic analysis of tumor cells from HGSOC PDX mouse models. **(A)** UMAP visualization of 28,422 cells with annotated cell types. **(B)** Proportions of identified cell types across treatment groups: control, pembrolizumab, and combination of pembrolizumab and anlotinib. **(C)** Dot plot showing expression of representative marker genes used for cell type identification. **(D)** UMAP plots separated by treatment group. **(E)** Feature plots displaying the expression patterns of key marker genes across all cells.

### Cell interaction demonstrate the impact of different treatment on cellular communication

To investigate the impact of different treatments on cell interactions among various cell types, we used CellChat to compare interactions across three treatment groups. Our analysis revealed that pembrolizumab enhanced interactions between lymphocytes, including CD8+/NK and CD4+ T cells, and epithelial cells. In the pembrolizumab and anlotinib combination group, interactions between CD8+/NK cells and epithelial cells were further strengthened, and the regulatory effect of monocytes on epithelial cells was also increased (**Figure 2A**).

**Figure 2:**
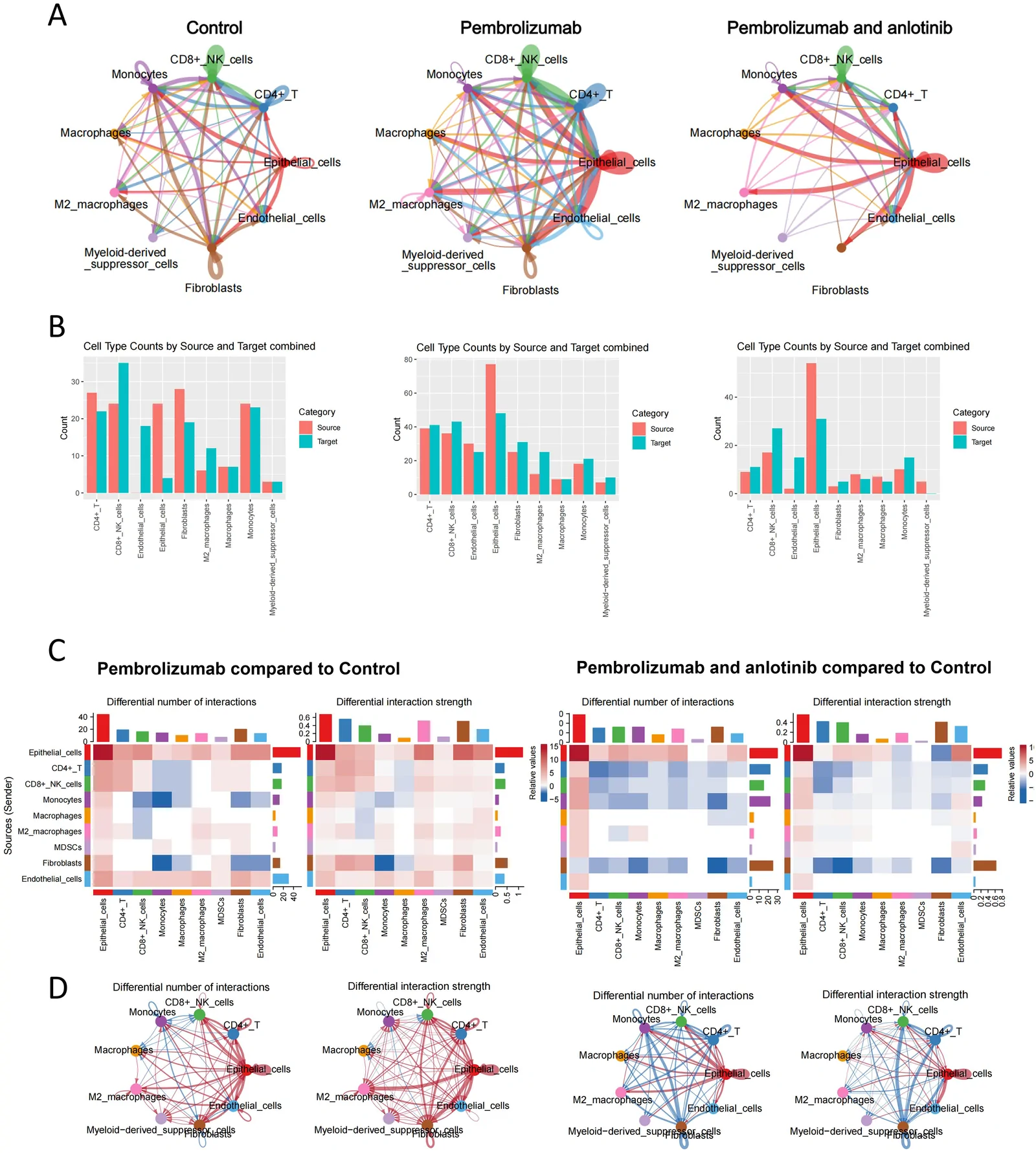
CellChat analysis of major cell types across treatment groups. **(A)** CellChat analysis of intercellular communication among major cell types under different treatment conditions. **(B)** Bar plot showing the number of signaling interactions (as source and target) for each cell type across treatment groups; bar plots show descriptive cell-type proportion changes from scRNA-seq data; limited valid biological replicates preclude formal inter-group statistical testing. **(C)** Comparison of total interaction numbers between the pembrolizumab group vs. control and the pembrolizumab plus anlotinib group vs. control. **(D)** Comparison of both the number and strength of differential interactions between the pembrolizumab group and control, and between the combination group and control.

Next, we compared the cell type counts by source and target across the three treatment groups. In the pembrolizumab group, CD4+ T cells were more frequently targeted than being a source, suggesting that pembrolizumab may induce regulation of CD4+ T cells. In the combination group of pembrolizumab and anlotinib, CD4+ and CD8+/NK cells were predominantly targets rather than sources, indicating a more substantial regulatory effect on these cells compared to the control group (**Figure 2B**).

We further compared interactions between the pembrolizumab and control groups, as well as the combined treatment and control groups. Both treatment groups showed enhanced epithelial cell interactions as targets. Notably, in the pembrolizumab and anlotinib combination group, interactions between monocytes and CD8+/NK cells (as sources) and epithelial cells (as targets) were markedly increased, suggesting that these cell types may play a pivotal role in the efficacy of the combined treatment (**Figures 2C, D**).

### Myeloid cells subcluster and interaction

To further explore the mechanism underlying the enhanced interaction between monocytes and epithelial cells in the combination treatment group, we performed subcluster analysis of myeloid cells (**Figure 3A**). A total of nine subclusters were identified, and their marker genes are presented in the dot plot (**Figure 3B**). We then compared the distribution of these subclusters across the three treatment groups and found that clusters 1 and 7 were primarily composed of monocytes, while cluster 2 contained a mix of monocytes and macrophages (**Figure 3C**). Subsequent comparison of the proportions of clusters 1, 2, and 7 revealed that cluster 1 increased numerically in both the pembrolizumab and combination treatment groups, with the numerically highest increase observed in the combination group. In contrast, the proportion of cluster 2 decreased numerically in both treatment groups, especially in the combination group (**Figure 3D**). Next, we conducted subcluster analysis of epithelial cells, identifying eight distinct subclusters. We compared the distribution of these epithelial subclusters among the three treatment groups and observed notable differences (**Figure 3E**). Analysis of cell-cell interactions between monocyte subclusters and epithelial subclusters showed that in the combination treatment group, myeloid subcluster 7 exhibited active interactions with epithelial cells (**Figure 3F**). Notably, the GRN-SORT1 signaling pathway was upregulated in the combination group, particularly between myeloid subcluster 1, 7 and epithelial subclusters 0, 2, 3 (**Figure 3G**). Among all ligand-receptor pairs identified by CellChat, GRN-SORT1 exhibited one of the highest communication probabilities across multiple myeloid-epithelial subcluster pairs in the combination treatment group (**Figure 3H**). GRN-SORT1 was consistently enriched between myeloid subclusters 1 and 7 and epithelial subclusters 0, 2, and 3, indicating that it represents a prominent communication axis induced by combination therapy. Furthermore, feature plots showed complementary expression patterns, with GRN predominantly expressed in myeloid cells and SORT1 enriched in epithelial cells (**Figure 3I**), further supporting the biological relevance of this interaction.

**Figure 3:**
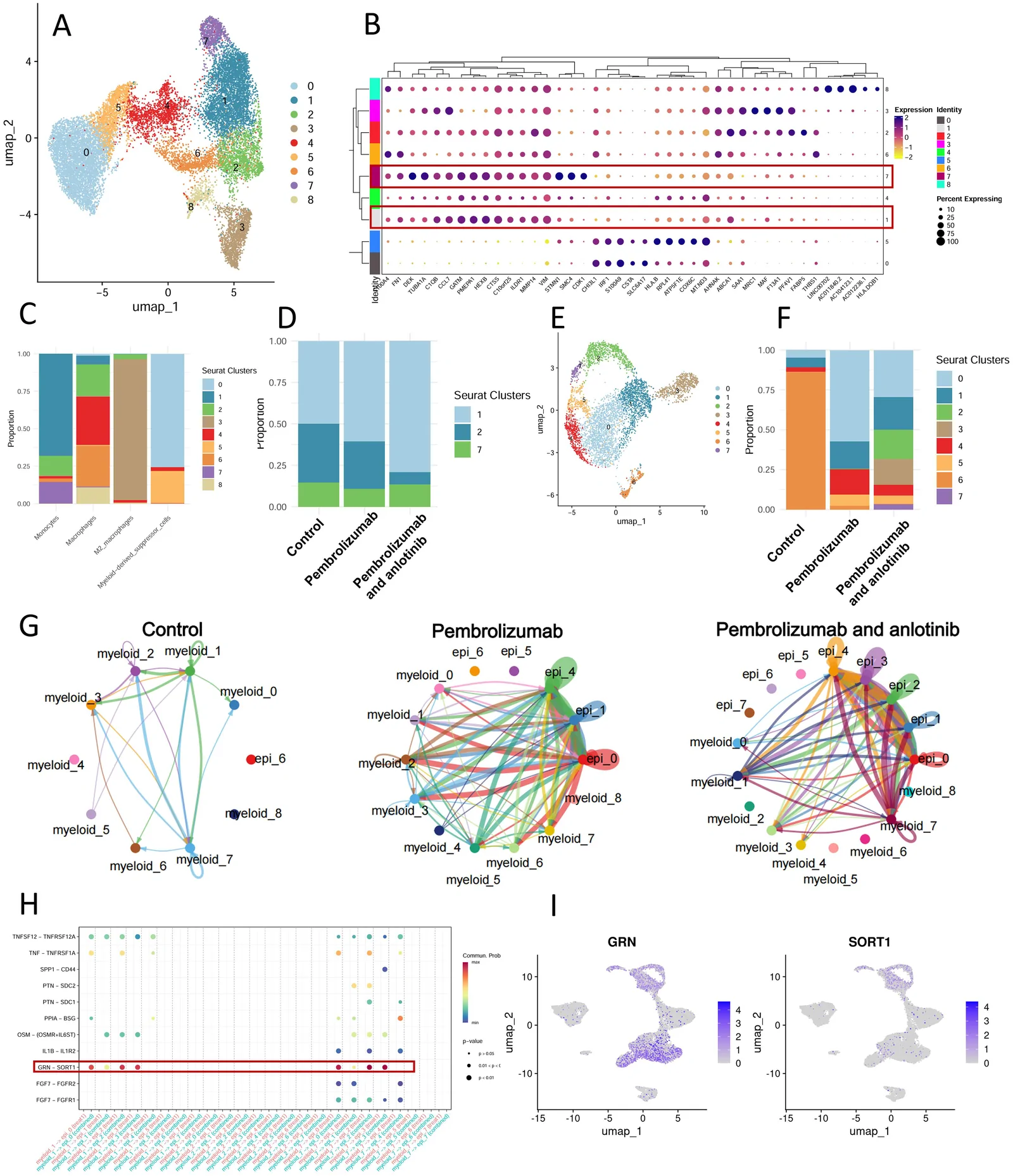
Subcluster analysis of myeloid and epithelial cells. **(A)** UMAP visualization of myeloid cell subclusters. **(B)** Dot plot showing expression of representative marker genes for each myeloid subcluster. **(C)** Annotation of major cell types corresponding to myeloid subclusters, along with their proportions. **(D)** Proportions of myeloid subclusters 1, 2, and 7 across different treatment groups; bar plots show descriptive cell-type proportion changes from scRNA-seq data; limited valid biological replicates preclude formal inter-group statistical testing. **(E)** UMAP visualization of epithelial cell subclusters. **(F)** Proportions of epithelial subclusters across treatment groups. **(G)** CellChat analysis of intercellular communication between selected myeloid and epithelial subclusters. **(H)** Dot plot illustrating communication probabilities between myeloid subclusters 1, 2, and 7 and epithelial subclusters. **(I)** Feature plots showing expression patterns of *GRN* and *SORT1*.

### Immune cells subcluster and interaction

The noticeable differences in lymphocyte proportions prompted us to further analyze lymphocyte subclusters. Seven distinct clusters were identified, and marker genes for each cluster were calculated (**Figures 4A, B**). To assess how different treatments influence immune cell interactions, we compared the interactions between lymphocyte subclusters and myeloid subclusters across the three treatment groups. In the combination treatment group, enhanced interactions were observed between myeloid subcluster 7 and lymphocyte subclusters 0 and 2 (**Figure 4C**). Notably, the CCL7–CCR1 signaling pathway was activated between myeloid subcluster 7 and lymphocyte subcluster 0 in the combination group (**Figure 4D**). Expression analysis confirmed that CCL7 was primarily expressed by myeloid cells, while CCR1 was predominantly expressed by lymphocytes (**Figure 4E**). Lymphocyte subcluster 0 mainly consisted of CD8^+^/NK T cells (**Figure 4F**). There were no significant differences in the proportions of lymphocyte subclusters across the three treatment groups (**Figure 4G**). To further explore whether the combination treatment enhanced immune activation, we compared the proportion of regulatory T cells (Tregs) between the groups. The Treg proportion was numerically lower in the combination treatment group compared to both the control and pembrolizumab-only groups (**Figure 4H**).

**Figure 4:**
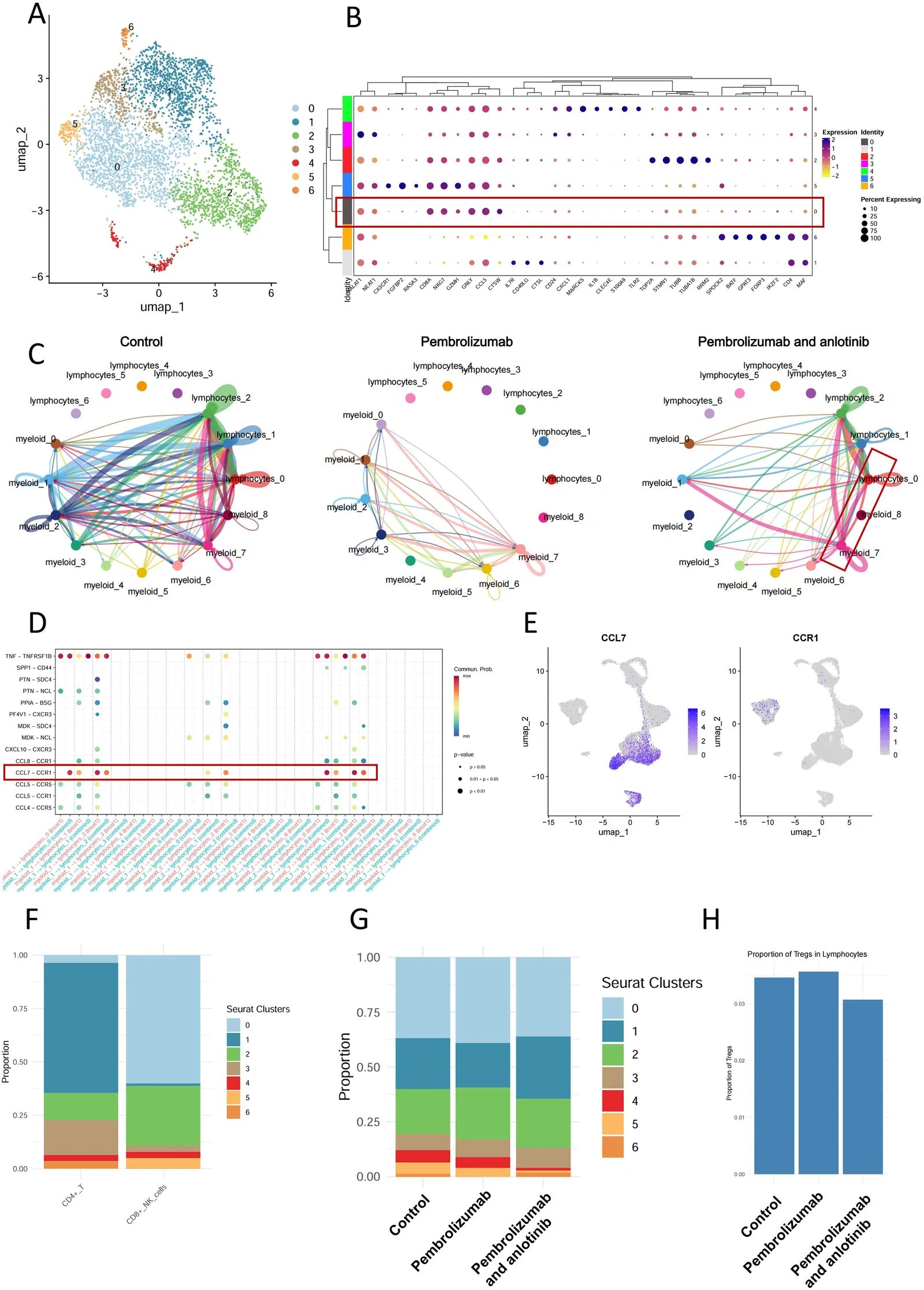
Subcluster analysis of lymphocytes. **(A)** UMAP visualization of lymphocytes subclusters. **(B)** Dot plot showing expression of representative marker genes for each lymphocyte subclusters. **(C)** CellChat analysis of intercellular communication between selected myeloid and lymphocytes subclusters. **(D)** Dot plot illustrating communication probabilities between myeloid subclusters 1, 2, and 7 and lymphocytes subclusters. **(E)** Feature plots showing expression patterns of *CCL7* and *CCR1*. **(F)** Annotation of major cell types corresponding to lymphocytes subclusters, along with their proportions. **(G)** Proportions of lymphocytes subclusters across different treatment groups. **(H)** Proportions of Tregs across different treatment groups; bar plots show descriptive cell-type proportion changes from scRNA-seq data; limited valid biological replicates preclude formal inter-group statistical testing.

### Combined pembrolizumab and anlotinib therapy targets epithelial subclusters and enhances immune interactions via GRN-SORT1 and CCL7–CCR1 signaling

To investigate the regulatory effects on epithelial clusters, we performed pathway analysis of epithelial cell subclusters (**Supplementary Figure 4**). Among all epithelial subclusters, the proportion of subcluster 6 showed the most significant decrease in the combination treatment group compared to the control group, suggesting that this subcluster was a primary target of pembrolizumab and anlotinib combined therapy (**Figure 5A**). Pathway analysis further revealed that the VEGF signaling pathway was downregulated in epithelial subcluster 6 compared to other subclusters, suggesting a potential link between low VEGF pathway activity and increased sensitivity to the combined treatment (**Figures 5B, C**).

**Figure 5:**
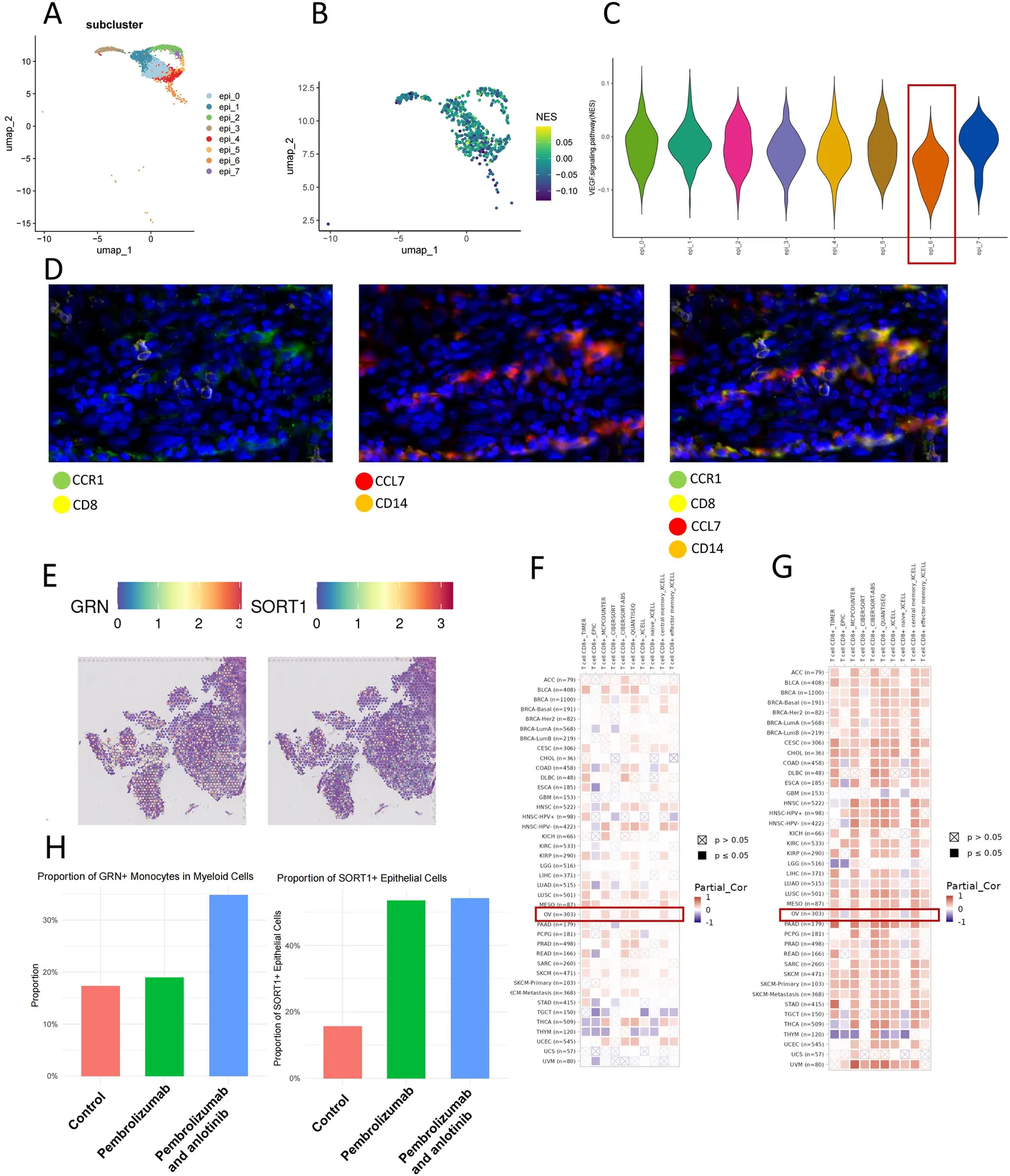
Validation of interactions between myeloid cells with epithelial cells and lymphocytes via GRN-SORT1 and CCL7-CCR1 signaling. **(A)** UMAP visualization of epithelial subclusters. **(B)** Featureplot showing the VEGF pathway expression in epithelial cells. **(C)** Violin plot showing the VEGF pathway expression in epithelial cell subclusters. **(D)** Spatial co-localization of CD14^+^CCL7^+^ monocytes and CD8^+^CCR1^+^ T cells; representative qualitative immunofluorescence images are shown; quantitative colocalization analysis was not performed in this study. **(E)** Spatial plot showing co-localization of GRN and SORT1 expression. **(F)** Correlation between CCL7 expression and CD8^+^/NK T cell infiltration. **(G)** Correlation between CCR1 expression and CD8^+^/NK T cell infiltration. **(H)** Proportion of GRN^+^ monocytes and SORT1^+^ epithelial cells across different treatment groups.

To validate the involvement of the CCL7–CCR1 signaling pathway between monocytes and CD8^+^/NK T cells, we conducted multicolor immunofluorescence to examine interactions between CD14^+^CCL7^+^ monocytes and CD8^+^CCR1^+^ T cells. This analysis revealed close spatial proximity and interaction between these two cell types in HGSOC patient samples (**Figure 5D**). Of note, no quantitative colocalization metrics were calculated for this dataset. To assess the spatial context of GRN-SORT1 interactions, we analyzed publicly available spatial transcriptomics data, which demonstrated co-localization of GRN and SORT1 expression (**Figure 5E**).

Using TIMER 2.0, we examined correlations between CCL7, CCR1 and CD8^+^ T cell infiltration in various cancer types. CCL7 and CCR1 expression were positively correlated with CD8^+^ T cell infiltration (**Figures 5F, G**). These findings suggest that targeting the GRN-SORT1 axis may enhance the efficacy of pembrolizumab and anlotinib combination therapy in HGSOC. Furthermore, the combination treatment may promote CD8^+^/NK T cell infiltration through CCL7^+^ monocytes.

## Discussion

Our study provides insights into the immune landscape of HGSOC under pembrolizumab and anlotinib combination therapy using a humanized PDX model. By applying single-cell RNA sequencing, we observed significant shifts in immune cell proportions across treatments, particularly in lymphocytes and myeloid cells. The increase in CD4+ cell populations and the decrease in Treg cells in the combination therapy group are consistent with potential immune-activating effects.

Cell-cell interaction analysis further revealed that the combination of pembrolizumab and anlotinib was associated with enhanced GRN-SORT1 signaling activity between monocytes and epithelial cells. Among these interactions, myeloid subcluster 1, 7 and epithelial subclusters 0, 2, 3 demonstrated the most pronounced activity in the combination group compared to the control. Sortilin (SORT1) has been reported to facilitate EGFR internalization from the plasma membrane (26), and its loss in tumor cells has been shown to enhance cell proliferation by sustaining EGFR signaling at the cell surface. In our study, we observed that GRN-expressing monocytes exhibited active interactions with sortilin-expressing epithelial cells specifically in the pembrolizumab and anlotinib combination treatment group. This finding suggests the possibility that the combined therapy may, at least in part, act through sortilin signaling in epithelial cells, potentially infuencing EGFR internalization or degradation and modulating downstream oncogenic signaling (26). Notably, epithelial cluster 6, which was significantly reduced in the combination group relative to both the control and pembrolizumab-only groups, showed a marked downregulation of VEGF signaling compared to other epithelial clusters. Previous studies have found that VEGFR-2 inhibitor and VEGFR-2 inhibitor combined with erlotinib both significantly decreased cell viability in erlotinib-resistant and EGFR-mutated cell lines (27). This result suggest that suppress VEGF signaling could also suppressing EGFR signaling and might explain our finding that one epithelial cluster exhibited downregulation of VEGF signaling barely existed after combination therapy treatment. These findings suggest that the pembrolizumab and anlotinib combination therapy is associated with a reduction in epithelial cells with low VEGF pathway activity. Given that both EGFR and VEGFR induce activation of signal transduction pathways that regulate cellular growth and proliferation and the two signaling pathways can function exclusively to sustain tumor growth (28–30), anlotinib may suppress VEGF signaling pathway and it is hypothesized that it could also influence EGFR signaling pathway through the GRN-SORT1 signaling and the internalization of EGFR.

Chemokine (C-C motif) ligand 7 (CCL7) is a chemotactic factor expressed in mononuclear cells, endothelial cells and fibroblasts. Its receptor CCR1 is expressed on multiple cell types, including T cells and antigen-presenting cells (APCs) such as monocytes, macrophages, and dendritic cells (DCs) (31). In this study, we also found the interaction between monocytes and CD8^+^/NK T cells particularly through CCL7–CCR1 signaling. To validate the interaction between CCL7^+^ monocytes and CCR1^+^ CD8^+^/NK T cells, we did multicolor immunofluorescence and confirmed the co-localization CCL7^+^CD14^+^ monocytes and CCR1^+^CD8^+^ T cells. Moreover, TIMER 2.0 analysis demonstrated a positive correlation between CCL7, CCR1 expression, and CD8+ T cell infiltration in HGSOC and other cancers, reinforcing the notion that these pathways may contribute to the efficacy of pembrolizumab and anlotinib combined therapy.

Owing to limited valid biological replicates for single-cell RNA-seq, all cell proportion trends observed across treatment groups are merely numerical descriptive findings without formal statistical intergroup comparisons. The spatial relationship between CD14^+^CCL7^+^ monocytes and CD8^+^CCR1^+^ T cells was only assessed via qualitative multicolor immunofluorescence observations, without quantitative colocalization analysis. Although representative images reveal their close spatial proximity, further studies incorporating quantitative colocalization metrics and larger patient cohorts are required. In this study, PBMCs obtained from a single healthy donor (purchased from MileCell Biotechnology) were used to construct the humanized PDX model for proof-of-concept investigation of the therapeutic synergy between pembrolizumab and anlotinib. It should be noted that the use of a single patient-derived tumor model and a single PBMC donor limits the generalizability of our findings, given the intrinsic heterogeneity of HGSOC and the inter-donor variability in immune reconstitution. Nonetheless, our results suggest a mechanistic rationale for the combination regimen and support the necessity of future clinical validation.

Given these findings derived from single-cell transcriptome and spatial transcriptome analysis, pembrolizumab and anlotinib combined therapy may be associated with recruitment CD8^+^/NK T cells by CCL7^+^ monocytes to activate the immune microenvironment, and may also involve GRN-expressing monocytes promoting EGFR internalization. GRN-SORT1 and CCL7-CCR1 signaling represent candidate axes that may contribute to the synergistic effect of pembrolizumab and anlotinib combined therapy; however, definitive functional validation is required in follow-up experimental studies to confirm their biological relevance.

## Data Availability

The matrix data that support the findings of this study have been deposited in the Figshare repository and are publicly available at: DOI: 10.6084/m9.figshare.33201873, https://figshare.com/articles/dataset/Data/33201873.
