# Supplementary material for: Synergistic antitumor effects of combined pembrolizumab and anlotinib via EGFR inhibition and tumor microenvironment remodeling in high-grade serous ovarian cancer
Source: Front Oncol. 2026 Oct 19;16:1880902. doi: 10.3389/fonc.2026.1880902 (PMC13724453; doi:10.3389/fonc.2026.1880902)
Supplement: Supplementary file 1 [file Image1.pdf]

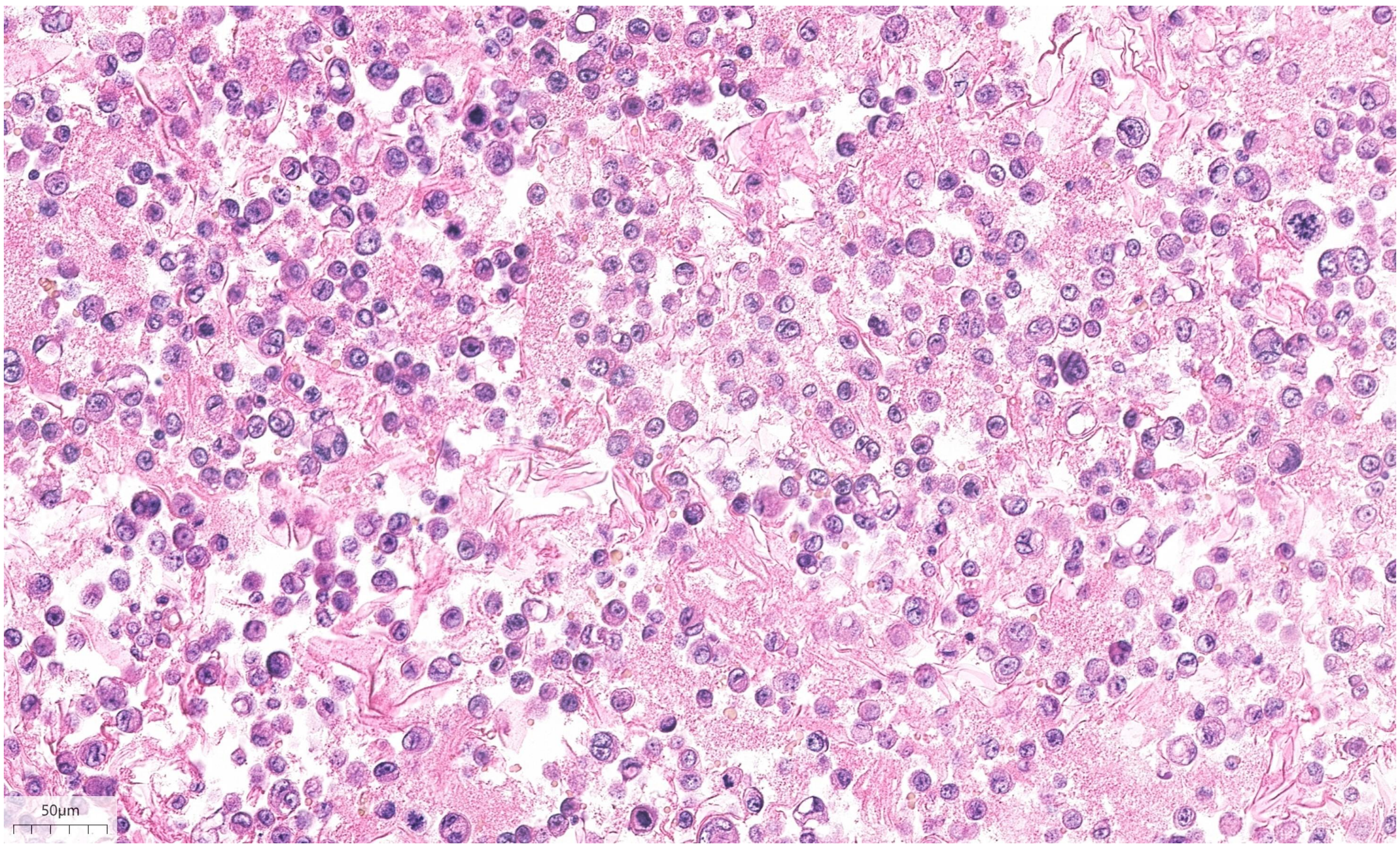

Supplementary Figure 1. Histopathological confirmation of HGSOC(×40).

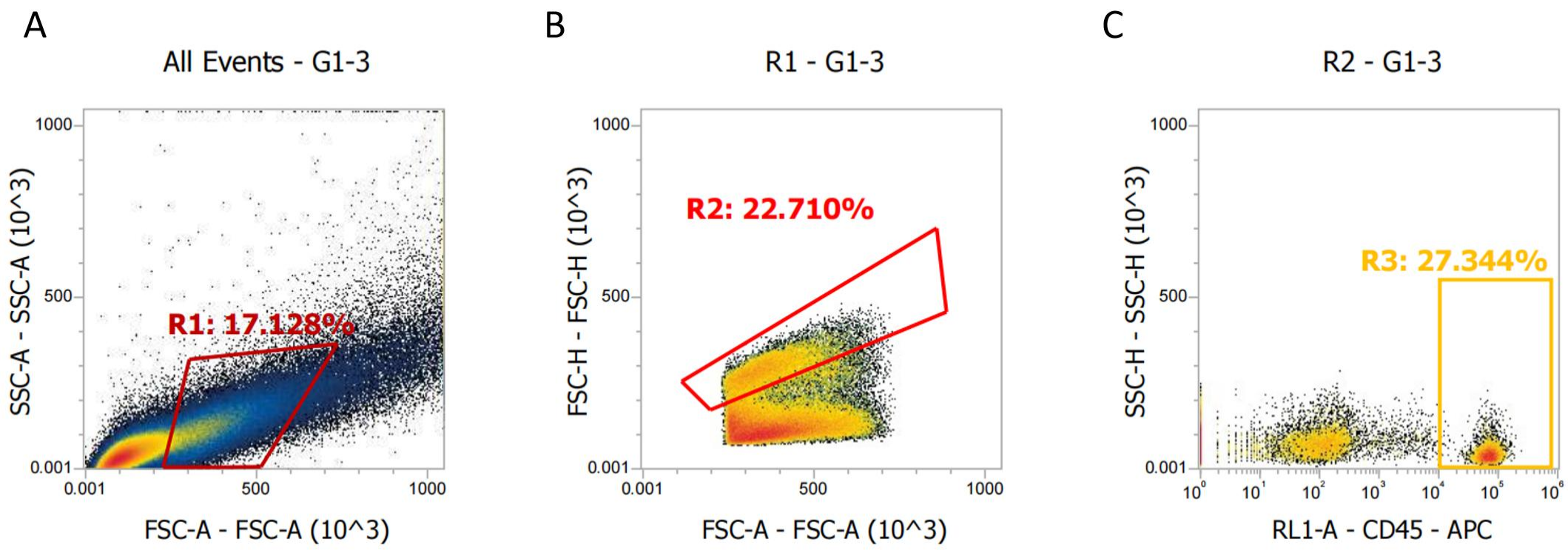

Supplementary Figure 2. Gating strategy for human CD45+ cell engraftment.

Representative flow cytometry plots illustrating the sequential gating of peripheral blood from humanized PDX mice: (A) exclusion of debris via FSC-A vs SSC-A (R1); (B) doublet discrimination using FSC-H vs FSC-A (R2); and (C) identification of human CD45+ immune cells by APC-CD45+ staining (R3). Human $CD45^+$ cells accounted for 21.61% ± 7.73% of total live cells in the peripheral blood, n = 5 mice per group.

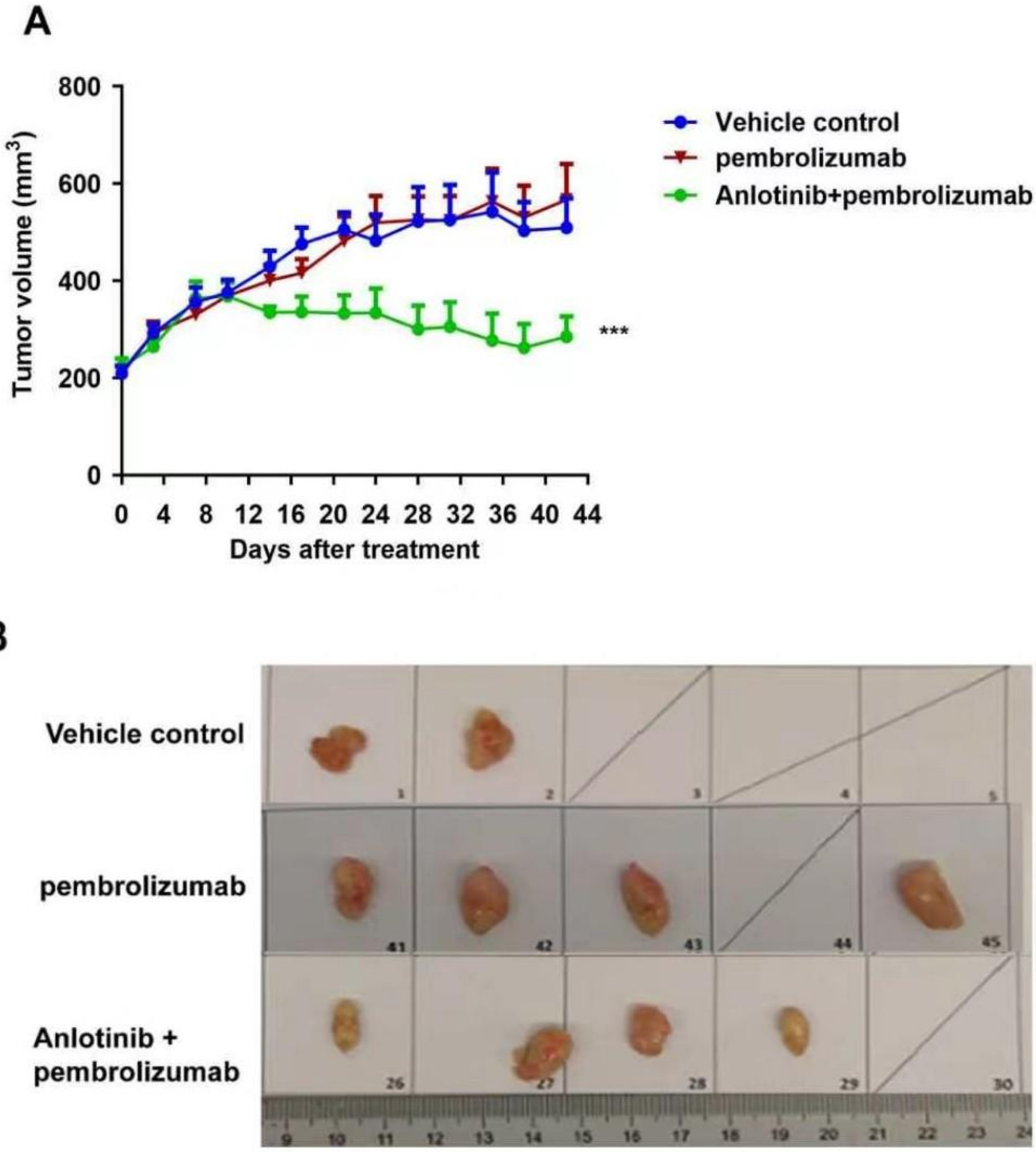

Supplementary Figure 3. Effect of pembrolizumab and anlotinib on tumor growth in PDX models.

(A) Tumor growth curves showing the volume of tumors over time in NSG mice treated with vehicle control, pembrolizumab monotherapy, or a combination of anlotinib plus pembrolizumab. Tumor volumes were measured every 4 days following the initiation of treatment. The combination therapy group exhibited a significant reduction in tumor volume compared to the other groups. Data are presented as mean ± SEM. Tumor volumes were compared using two-way repeated-measures ANOVA with Bonferroni correction for multiple comparisons. *$P < 0.05$, **$P < 0.01$, ***$P < 0.001$ versus control.

(B) Representative images of excised tumors from each treatment group at the end of the study (Day 42). Tumors from the anlotinib plus pembrolizumab group were noticeably smaller compared to those from the vehicle control and pembrolizumab monotherapy groups.

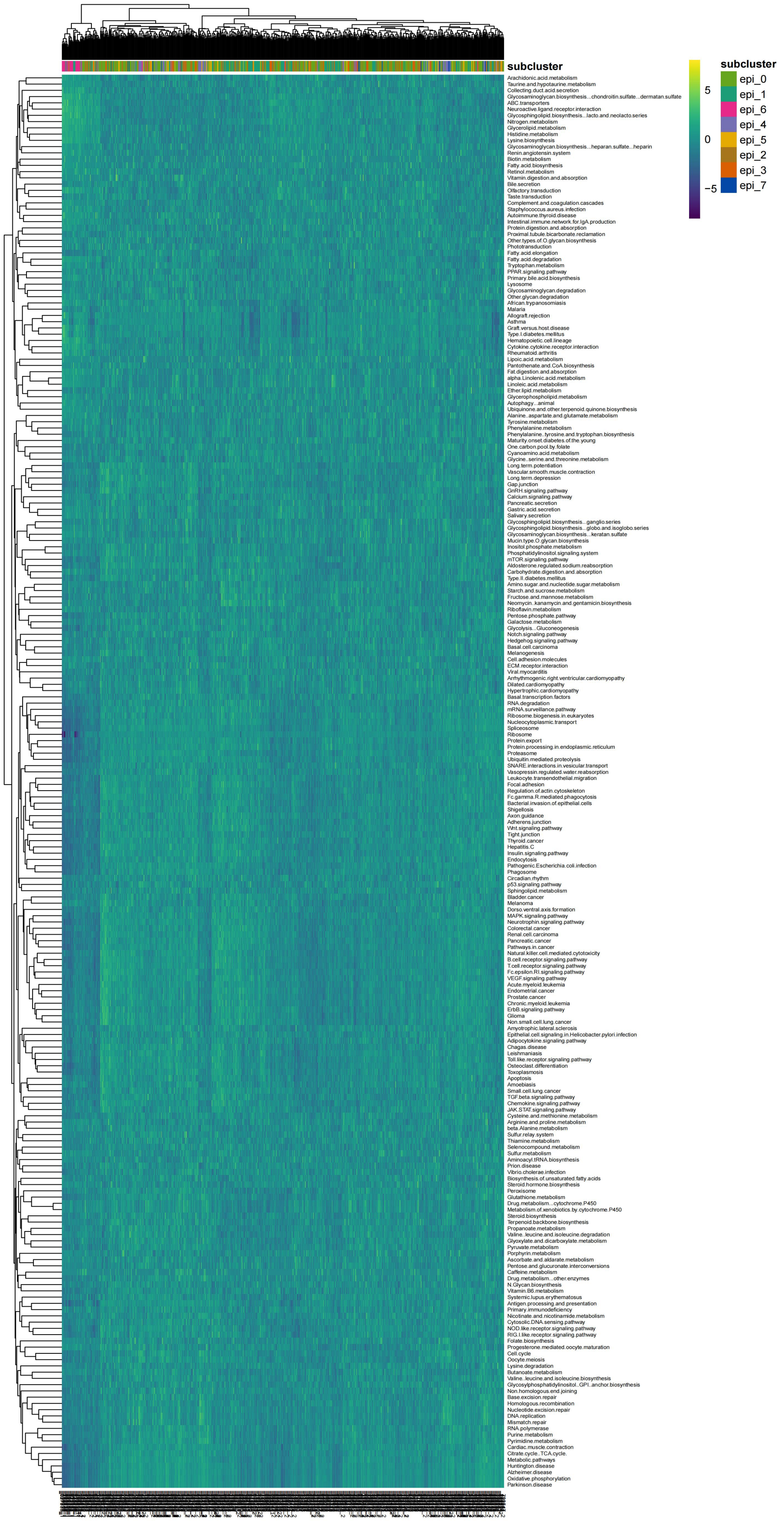

Supplementary Figure 4. Pathway enrichment analysis of epithelial cell subclusters.
