# Supplementary material for: Synergistic antitumor effects of combined pembrolizumab and anlotinib via EGFR inhibition and tumor microenvironment remodeling in high-grade serous ovarian cancer
Source: Front Oncol. 2026 Oct 19;16:1880902. doi: 10.3389/fonc.2026.1880902 (PMC13724453; doi:10.3389/fonc.2026.1880902)
Supplement: Supplementary file 2 [file Table1.docx]

**Supplementary Table 1. Clinicopathological and genomic characteristics of the PDX tumor donor**

| Category | Detailed Information |
| --- | --- |
| BRCA mutation status | Matched ovarian tumor tissue, malignant ascites, and peripheral blood samples were all subjected to targeted next-generation sequencing, with BRCA1 and BRCA2 analyzed for point mutations, insertions, and deletions using the same approach, and no pathogenic alterations were identified. |
| HRD status | Not assessed. |
| Prior Treatment History | The first-line regimen consisted of paclitaxel plus carboplatin for 10 cycles; the patient showed progression disease after 4 months. Then second-line treatment with olaparib (300 mg twice daily) was administered for 1.5 month but was discontinued due to intolerance and progressive disease. Subsequently, the regimen was switched to a combination of pemetrexed, cisplatin, and bevacizumab. After two cycles, the patient showed stable disease. |
| Histopathological Diagnosis | High-grade serous ovarian carcinoma (HGSOC). Confirmed by hematoxylin and eosin (H&E) staining of the resected bilateral ovaries and fallopian tubes, revealing poorly differentiated carcinoma with complex papillary/solid architecture, marked nuclear pleomorphism, and slit-like fenestrations. Involvement of uterine serosa and multiple lymph node metastases was observed. Diagnosis further corroborated by cytological examination of malignant ascites. |
